# Longitudinal Change in Brain Functional Connectivity with Herpes Zoster Patients: Neuroimaging Case Series

**DOI:** 10.3390/medicina59061045

**Published:** 2023-05-29

**Authors:** Changjae Kim, Joongbaek Kim, Hyunjae Chang, Dakyung Hong, Sanghyun Hong, Hosik Moon

**Affiliations:** Department of Anesthesiology and Pain Medicine, College of Medicine, The Catholic University of Korea, Seoul 03312, Republic of Korea

**Keywords:** herpes zoster, postherpetic neuralgia, chronic pain, magnetic resonance imaging, neural pathways, prefrontal cortex, functional, connectivity, functional neuroimaging

## Abstract

The exact mechanism involved in the development of postherpetic neuralgia (PHN) is not yet known. The objective of this study was to evaluate longitudinal functional connectivity (FC) changes in the neuroimaging case series of patients with acute herpes zoster (HZ). Cases: This study included five patients who had symptoms of HZ. Functional magnetic resonance imaging was conducted at enrollment and 3 months to determine FC changes. Of the five patients, three developed PHN. In the PHN subjects, the FC of the left superior frontal gyrus (SFG) and the right inferior frontal gyrus (IFG) were activated. The left SFG is known to contribute to higher cognitive functions and working memory. The right IFG is associated with pain processing and empathy for pain. Conclusions: Although only a few patients were enrolled in this study, the PHN could be affected by pain itself, as well as pain memory and psychological aspects such as empathy for pain.

## 1. Introduction

Herpes zoster (HZ) is a viral infection in the cranial nerve ganglia or dorsal root ganglia. It is associated with the reactivation of the varicella zoster virus (VZV). The clinical features of HZ include skin and neuropathic symptoms. Most skin symptoms are recovered within a few weeks. Pain also gets better as skin symptoms improve. However, in some cases, severe pain could be prolonged for a very long period, which is called postherpetic neuralgia (PHN). The exact mechanism of developing chronic pain is not yet known. Genetic mechanisms, priming effects on a cellular level, and brain functional connectivity (FC) changes are considered as factors contributing to chronic pain [1].

Functional magnetic resonance image (fMRI) is a non-invasive, objective, and useful research tool to analyze the brain functionally and structurally [2]. FC analyzed by fMRI has the potential to be a biomarker for acute and chronic pain. It can help identify the mechanism of chronic pain. Several fMRI studies about neurologic mechanisms that make HZ become PHN were published [3,4]. However, studies on longitudinal changes of FC from HZ to PHN have not yet been reported. Thus, the objective of this study was to evaluate longitudinal FC changes in the neuroimaging case series of patients with acute HZ. Although this is not a well-controlled study, this case series can provide useful knowledge of how HZ becomes chronic.

## 2. Case Report

This case study included five patients diagnosed with HZ who visited the pain clinic at a university hospital from July 2018 through December 2018. They participated in the study for three months after enrollment. The Institutional Review Board of the Catholic University of Korea approved protocols for this study (approval number: SC18OES10042). This study was conducted in accordance with the Declaration of Helsinki. All patients provided written informed consent to participate in this study.

All subjects were adults aged more than 40 years. Their visits to the pain clinic were within 14 days after a manifestation of pain and skin symptoms, including rash, maculopapular, vesicle lesion, and so on, on the dermatome of the right chest or right abdomen (Table 1). Before HZ treatments, the numeric rating scale (NRS) (0, no pain; 10, the worst pain imaginable), Douleur neuropathique 4 (DN4), Beck depression inventory (BDI), short-form McGill pain questionnaire (SF-MPQ), current perception threshold (CPT), and brain fMRI were evaluated. All received active treatments, including nerve blocks such as paravertebral block, intercostal nerve block, epidural block, and so on, using local anesthetics and/or corticosteroids and medications including gabapentin, pregabalin, and tramadol. The frequency of the nerve blocks and the dosage of the medications depended on the symptoms of the patient. Three months after the appearance of skin symptoms, the above examination was performed again. Among five subjects, three (Cases 3, 4, and 5) developed PHN in a 3-month period (Table 1). In this study, there was a correlation between the development of PHN and DN4 (0-month) (Spearman’s ρ = 0.408, *p* = 0.044).

**Figure 1:**
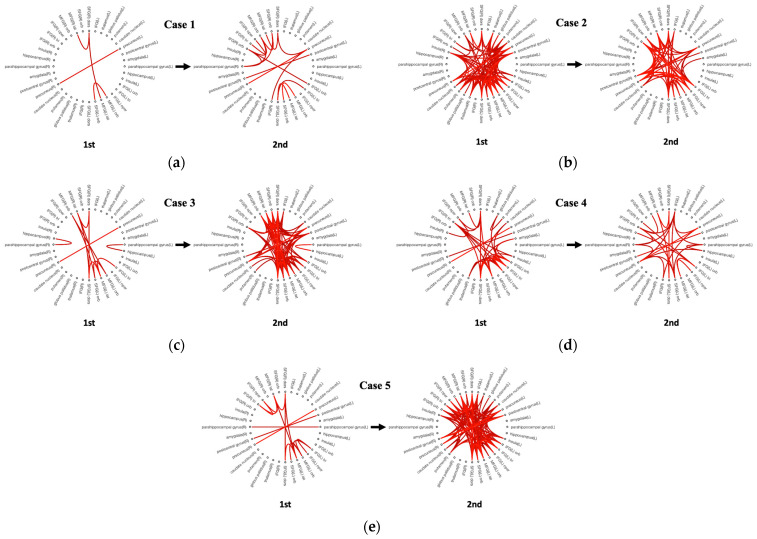
Connectograms of the 36 ROIs selected. (**a**) Case 1, (**b**) Case 2, (**c**) Case 3, (**d**) Case 4, and (**e**) Case 5. The red solid line in each connectogram reflects the function relationship between both ROIs (r > 0.6 or r < 0.6, Pearson’s correlation coefficient). ROIs, regions of interests. For the abbreviation of brain regions, please refer to Table 2.

The fMRI study was conducted at a resting state without any experimental tasks to investigate the FC. The subjects underwent functional imaging using a 1.5 T MRI scanner (Siemens, Erlangen, Germany). The functional images were acquired using a T2*-weighted single-shot echo-planar imaging (EPI) sequence that covered the whole brain (TE, 30 ms; TR, 3000 ms; flip angle, 90°; slice thickness, 3 mm without a gap; matrix, 64 × 64 mm; FOV, 192 mm) to obtain the blood-oxygen-level-dependent (BOLD) contrast. The total scan time was 10 min. Raw EPI data excluding the first five volumes (i.e., dummy volumes to allow any T1-effect to equilibrate) were preprocessed using SPM12 (www.fil.ion.ucl.ac.uk/spm) with default parameters in the following order: head motion correction, co-registration, segmentation, normalization on to the Montreal Neurological Institute coordinates with a 2 mm isotropic voxel size, and spatial smoothing using an 8 mm isotropic full-width at half-maximum Gaussian kernel. Each voxel was assigned to the parcellation of regions of interest (ROIs) based on masks defined by an Automated Anatomical Labeling (AAL) [5]. We selected a total of 36 ROIs (Table 2) based on relevant studies [3,4,6]. The FC of different ROIs was determined for every single subject based on a bivariate correlation method (r > 0.6 or r < 0.6, Pearson’s correlation coefficient) (Figure 1). In the recovery group of patients who were recovered from AHZ at three months, the commonly activated FCs among ROIs compared to the initial FC were postcentral gyrus (L)–postcentral gyrus (R) and IFG (R) (opercular part)–IFG (R) (triangular part). In the PHN group of patients who had suffered from pain at three months, the common activated FC compared to one month was SFG (L) (orbital part)–IFG (R) (orbital part) (Table 3). Additionally, intra-hemispheric and inter-hemispheric connectivity seemed to be different between the recovery group and the PHN group, although statistical analysis was not possible (Table 4).

Recovery group: patients who were recovered from acute herpes zoster at three months; PHN group: patients who had suffered from pain at three months. Suppression means the common suppressed FC at 3 months compared to that at 1 month within each group. No change meant that there was no significant difference between 1 month and 3 months within each group. Activation meant the commonly activated FC at 3 months compared to 1 month within each group. FC, functional connectivity; R, right; L, left; PHN, postherpetic neuralgia. For abbreviations of the brain regions, please refer to Table 2.

The intra-hemispheric connectivity means the connectivity within each hemisphere. The inter-hemispheric connectivity means the connectivity between each hemisphere.

## 3. Discussion

In this study, we investigated longitudinal changes in the FC of PHN patients and compared them with those of patients who had not developed PHN. We found that the postcentral gyrus (L)–postcentral gyrus (R) and the IFG (R) (opercular part)–IFG (R) (triangular part) were commonly activated FCs in the recovery group, while the SFG (L) (orbital part)–IFG (R) (orbital part) was commonly activated in the PHN group.

Jiang et al. found the FC in the dorsolateral prefrontal cortex (PFC) and precuneus/posterior cingulate cortex (PCC) is decreased in PHN patients [6]. Fu et al. found that FCs between the dorsolateral and superficial amygdala, as well as several regions, including the temporal lobe and frontal lobe, are reduced, whereas FCs between the superficial amygdala and the precentral cortex, as well as the parietal lobe, are increased in PHN patients [7]. The precuneus and PCC maintain a highly active state while gathering internal and external information, which is crucial for processing sensory information [8]. By FC disruption, the amygdala could lose its ability to make a prudent decision by avoiding a dangerous one and to direct behavior to the goal [9]. The previous studies imply that PHN not only gives pain to patients but also causes functional impairment for organizing painful stimuli and the response to them, thus, making patients suffer more from the disease [6,7,8,9].

Baliki et al. performed a whole-brain connectivity analysis in rodents exposed to neuropathic injury and observed changes in the FC between limbic systems and between the nociceptive network and limbic systems for up to 4 weeks [10]. Bilbao et al. reported that the FC of the PFC and hippocampus is decreased in chronic pain patients [11]. Mutso et al. conducted a study about FC changes in back-pain patients [12]. They found increased hippocampal connectivity in back-pain patients. Specifically, the patients suffering persistent subacute back pain showed larger decreases in hippocampal connectivity with the medial prefrontal cortex than recovered subacute back pain. It seems that the reorganization of processing between the hippocampus and the cortex contributes to the transition from subacute pain to chronic pain [12].

In these case reports, commonly decreased FC did not exist among PHN patients. However, newly activated FC was commonly seen between the SFG (L) (orbital part) and the IFG (R) (orbital part) from the PHN group. The SFG (L) is known to contribute to higher cognitive functions, particularly working memory [13]. IFG (R) is associated with pain processing and empathy for pain [14]. Thus, neuroplasticity could support the view that as the pain becomes chronic, there is a progressive shift from pain to emotional networks [15]. Our findings indicate that PHN could be affected by pain memory and emotional factors for pain as well as the pain itself.

In the previous studies, inter-hemispheric connectivity tends to be decreased in patients who have painful diseases such as PHN or traumatic axonal injury than in healthy controls [6]. The possible alterations of structure and functions might lead to this pattern. This different pattern of inter-hemispheric connectivity can be utilized as a diagnostic marker for PHN [6]. In our data, a statistically valid tendency between the recovered group—not the healthy control group—and the PHN group was not found. However, two out of three patients who developed PHN showed evident growth of inter-hemispheric connectivity after three months from enrollment. According to previous studies, this inter-hemispheric connectivity growth is expected to decrease [6]. A follow-up study focusing on the further fluctuation of inter-hemispheric connectivity would be helpful to investigate and evaluate the prognosis of PHN.

This study has a few limitations. First, this study had a relatively small sample size. Thus, a larger sample is needed to confirm our results. Second, sticking to the concept of the modulation of the brain network and neuronal plasticity, the follow-up acquisition of fMRI for PHN patients could be useful for evaluating the effect of PHN on FC changes that might show another tendency. However, we did not have any further data in this study.

## 4. Conclusions

The development of PHN could be affected by pain memory, psychological and emotional aspects such as empathy for pain, and working memory, as well as pain itself. Thus, prolonged pain conditions can jeopardize a patient’s recovery.

## Figures and Tables

**Table 1:** Demographic characteristics of the five subjects.

		Case 1	Case 2	Case 3	Case 4	Case 5
Age, yr		54	71	44	65	54
Sex, M/F		M	F	F	F	M
Location of lesion		Right T4	Right T5	Right T4	Right T6	Right T10
NRS	0-month	8	7	8	6	10
	3-month	0	0	2	2	3
DN4	0-month	5	5	9	8	7
	3-month	2	2	3	2	2
BDI	0-month	32	33	40	25	36
	3-month	31	29	39	23	36
SF-MPQ	0-month	44	51	61	44	56
	3-month	23	20	31	24	29
CPT, µA	0-month	13.6	22.1	14.8	16.0	20.8
	3-month	12.4	21.6	11.8	10.0	10.6
Development of PHN, Y/S		N	N	Y	Y	Y

NRS, numeric rating scale; DN4, Douleur neuropathique 4; BDI, Beck depression inventory; SF-MPQ, short-form McGill pain questionnaire; CPT, current perception threshold; PHN, postherpetic neuralgia; T, thoracic.

**Table 2:** List of selected ROIs and abbreviations used in this study.

Regions		ROIs
Central region		
		Postcentral gyrus
Frontal lobe		
	Lateral surface	Superior frontal gyrus, dorsolateral (SFG_dors)
		Middle frontal gyrus, lateral (MFG_lat)
		Inferior frontal gyrus, opercular part (IFG_oper)
		Inferior frontal gyrus, triangular part (IFG_tri)
	Orbital surface	Superior frontal gyrus, orbital part (SFG_orb)
		Middle frontal gyrus, orbital part (MFG_orb)
		Inferior frontal gyrus, orbital part (IFG_orb)
Temporal lobe		
	Lateral surface	Inferior temporal gyrus (ITG)
Parietal lobe		
	Medial surface	Precuneus
Limbic lobe		
		Hippocampus
		Parahippocampal gyrus
Insula		
		Insula
Subcortical gray nuclei		
		Amygdala
		Caudate nucleus
		Putamen
		Globus pallidus
		Thalamus

ROIs, regions of interest.

**Table 3:** Common brain FC within each group.

Cases	Suppression	No Change	Activation
Recovery group (1, 2)	N/A	SFG(L)_orb–MFG(L)_orb Precuneus(L)–Precuneus(R) SFG(R)_dors–MFG(R)_lat	Postcentral gyrus(L)–Postcentral gyrus(R) IFG(R)_oper–IFG(R)_tri
PHN group (3, 4, 5)	N/A	SFG(L)_dors–SFG(R)_dors MFG(L)_lat–IFG(L)_tri Precuneus(L)–Precuneus(R) SFG(R)_dors–MFG(R)_lat	SFG(L)_orb–IFG(R)_orb

**Table 4:** Comparison of intra-hemispheric connectivity and inter-hemispheric connectivity in each subject.

	Case 1	Case 2	Case 3	Case 4	Case 5
Intra-hemispheric connectivity					
0-month, n	2	47	6	22	10
3-month, n	10	31	41	12	59
3-month–0-month, n	8	−16	35	−10	49
Inter-hemispheric connectivity					
0-month, n	2	43	6	13	7
3-month, n	5	24	37	13	48
3-month–0-month, n	3	−9	31	0	41

## Data Availability

The datasets used and/or analyzed in the current study are available from the corresponding author upon reasonable request.

## References

[1] Denk F., McMahon S.B., Tracey I. (2014). Pain vulnerability: A neurobiological perspective. Nat. Neurosci..

[2] Kang D.H., Son J.H., Kim Y.C. (2010). Neuroimaging studies of chronic pain. Korean J. Pain.

[3] Liu J., Gu L., Huang Q., Hong S., Zeng X., Zhang D., Zhou F., Jiang J. (2019). Altered gray matter volume in patients with herpes zoster and postherpetic neuralgia. J. Pain Res..

[4] Li J., Huang X., Sang K., Bodner M., Ma K., Dong X.W. (2018). Modulation of prefrontal connectivity in postherpetic neuralgia patients with chronic pain: A resting-state functional magnetic resonance-imaging study. J. Pain Res..

[5] Tzourio-Mazoyer N., Landeau B., Papathanassiou D., Crivello F., Etard O., Delcroix N., Mazoyer B., Joliot M. (2002). Automated anatomical labeling of activations in SPM using a macroscopic anatomical parcellation of the MNI MRI single-subject brain. Neuroimage.

[6] Jiang J., Gu L., Bao D., Hong S., He W., Tan Y., Zeng X., Gong H., Zhang D., Zhou F. (2016). Altered homotopic connectivity in postherpetic neuralgia: A resting state fMRI study. J. Pain Res..

[7] Fu W.-L., Tao W., Chen F.-Y. (2018). Altered Functional Connectivity of The Amygdala in Postherpetic Neuralgia. Prog. Biochem. Biophys..

[8] Hong S., Gu L., Zhou F., Liu J., Huang M., Jiang J., He L., Gong H., Zeng X. (2018). Altered functional connectivity density in patients with herpes zoster and postherpetic neuralgia. J. Pain Res..

[9] Kouneiher F., Charron S., Koechlin E. (2009). Motivation and cognitive control in the human prefrontal cortex. Nat. Neurosci..

[10] Baliki M.N., Chang P.C., Baria A.T., Centeno M.V., Apkarian A.V. (2015). CORRIGENDUM: Resting-state functional reorganization of the rat limbic system following neuropathic injury. Sci. Rep..

[11] Bilbao A., Falfan-Melgoza C., Leixner S., Becker R., Singaravelu S.K., Sack M., Sartorius A., Spanagel R., Weber-Fahr W. (2018). Longitudinal Structural and Functional Brain Network Alterations in a Mouse Model of Neuropathic Pain. Neuroscience.

[12] Mutso A.A., Petre B., Huang L., Baliki M.N., Torbey S., Herrmann K.M., Schnitzer T.J., Apkarian A.V. (2014). Reorganization of hippocampal functional connectivity with transition to chronic back pain. J. Neurophysiol..

[13] du Boisgueheneuc F., Levy R., Volle E., Seassau M., Duffau H., Kinkingnehun S., Samson Y., Zhang S., Dubois B. (2006). Functions of the left superior frontal gyrus in humans: A lesion study. Brain.

[14] Li Y., Li W., Zhang T., Zhang J., Jin Z., Li L. (2021). Probing the role of the right inferior frontal gyrus during Pain-Related empathy processing: Evidence from fMRI and TMS. Hum. Brain Mapp..

[15] Da Silva J.T., Seminowicz D.A. (2019). Neuroimaging of pain in animal models: A review of recent literature. Pain Rep..

